# Bioactive xanthones, benzophenones and biphenyls from mangosteen root with potential anti-migration against hepatocellular carcinoma cells

**DOI:** 10.1038/s41598-022-12507-8

**Published:** 2022-05-21

**Authors:** Siwattra Choodej, Kedkarn Koopklang, Achara Raksat, Natthaya Chuaypen, Khanitha Pudhom

**Affiliations:** 1 Department of Chemistry, Faculty of Science, Chulalongkorn University, Bangkok, 10330 Thailand; 2 Program in Biotechnology, Faculty of Science, Chulalongkorn University, Bangkok, 10330 Thailand; 3 Center of Excellence in Hepatitis and Liver Cancer, Department of Biochemistry, Faculty of Medicine, Chulalongkorn University, Bangkok, 10330 Thailand

**Keywords:** Chemical biology, Chemistry

## Abstract

Liver cancer refers primarily to hepatocellular carcinoma (HCC) accounting for over 90% of cases and is the highest incidence in men in Thailand. Over the past decades, the incidence of HCC dramatically increased with a strong rise of mortality rates. *Garcinia mangostana*, “Queen of Fruit” of Thailand, is known as a rich source of xanthones with potent cytotoxic properties against various cancer cells. Study on xanthones is provoking not only due to the structural diversity but also a wide variety of pharmacological activities. Hence the aim of the current study is to determine the effects of metabolites from *G. mangostana* root on cell proliferation and migration of hepatocellular carcinoma cells. Twenty-two metabolites, including two new benzophenones and one new biphenyl, were isolated and characterized. Five xanthones with a prenyl moiety showed significant cytotoxicity against both HCC cells tested; however, only dulxanthone D displayed the most promising activity on the migration of Huh7 HCC cells, comparable to sorafenib, a standard drug. Moreover, the compound dose-dependently induced apoptosis in Huh7 cells via mitochondrial pathway. Accordingly, dulxanthone D held a great potential for development as a novel migration inhibitor for effective HCC treatment.

## Introduction

Hepatocellular carcinoma (HCC) is number one deadliest disease in Thailand from the report of Ministry of Public Health. The risk factors for cancer are various, it could be from environment, life routine and human genetic^1,2^. Interestingly, carcinogenic infection from human papillomavirus (HPV), hepatitis B and C virus could lead approximately 25–40% to HCC even though the mechanism underlying progression still remains unclear. According to Thailand-based Cancer Registry reported, in 2015, liver cancer accounted for 15,912 patients out of 20,617 new cancer cases to deaths. Moreover, it occurs more often in men than women. Despite metastatic liver cancer being the most frequently seen type, for instance, spreading from breasts or colons, hepatocellular carcinoma (HCC) is also the most common primary cancer account for 80% of all primary liver cancers^3^. This is due to the typically late diagnosis with a median survival following diagnosis of approximately 6–20 months^4^. Currently, general drugs approved by the Food and Drug Administration (FDA) for liver cancer are extensively depends on patient’s symptoms. For examples, sorafenib tosylate, a kinase inhibitor drug is used with the HCC that cannot be removed by surgery^5,6^, and Bevacizumab, an angiogenesis inhibitor is given to patients who have not received systemic therapy or in metastatic stage^7^.

In addition, liver cancer has been grouping into five stages to prevent medical intervention harms in patients with cirrhosis. Liver cancer metastasis appears in fourth of five stages classify as advanced stage, indicating that the cancer could spread from origin to other organs^8^. For explanation, metastasis occurs when the primary cancer travels from its original place through the bloodstream and lymph node, then forms a secondary or metastatic tumor in the nearest organs. More importantly, HCC has a high recurrent rate of intrahepatic metastatic spread as well as extrahepatic metastasis to the colon^9,10^. Thus, this could estimate lessen of survival time in patient with around 11–13 months in nearly last stage. Nowadays, there are many treatments use to prevent cancer, such as immunotherapy, radiation therapy, or targeted therapy^11^. However, among various treatments, chemotherapy is the most often use to treat cancers. In addition, more than 50% of chemotherapy drugs are produced from natural compounds. This is due to a fewer toxicity and side-effects against normal cells^12^.

Mangosteen (*Garcinia mangostana* L.), a large perennial plant with lushes of leathery thick leaves, grown well and widely in tropical weather countries specially in Southeast Asia. It was known as ‘Queen of fruits’ due to the unique sweet-but-sour taste and the round, dark purplish appearance with thick green sepals arranged like a crown^13^. Additionally, not only a flavor that make mangosteen becomes well known but also because of its variety in medicinal applications. In many countries including Thailand, mangosteen has been used as a traditional medicine for recent past. Various parts from the mangosteen plant can be used in numerous ways. For instance, extracts of ripening fruits were used as diarrhea treatment whereas stem barks were used as wound infection treatment^14^. Nowadays, there are prevalent studies about metabolite compositions from mangosteen. The most common classes found are xanthones with α- and β-mangostin as major components. These compounds have been reported with many beneficial bioactivities such as anti-oxidation, anti-inflammatory, antimicrobial and anticancer^15^. Mangosteen also contains other class compositions, for examples, flavonoids, anthocyanins and benzophenones^16^.

Many previous studies have revealed bioactivities of xanthones especially with α-mangostin. Because of it being easily found, this largest constituent α-mangostin was used to study for medicinal usages. One of the common studies is anticancer activity. To demonstrate that, α-mangostin was applied with various cancer cell lines. As a result, α-mangostin could prevent and inhibit proliferation of various cancer cells such as colorectal, hepatocellular, and breast cancer cells^17^. Apart from α-mangostin and other xanthones, the study in bioactivity of other metabolites with anticancer activities are still lack and barely known. Although there were some reports, most were only preliminary investigation, as well as study on metabolites from mangosteen roots is still less. Because of this, the present study aims to isolate and characterize secondary metabolites from mangosteen roots and to study effect of these metabolites against HCC cell growth as well as their efficacy on cell migration (Supplementary Information).

## Results

### Isolation and identification of metabolites from mangosteen roots

**Figure 1:**
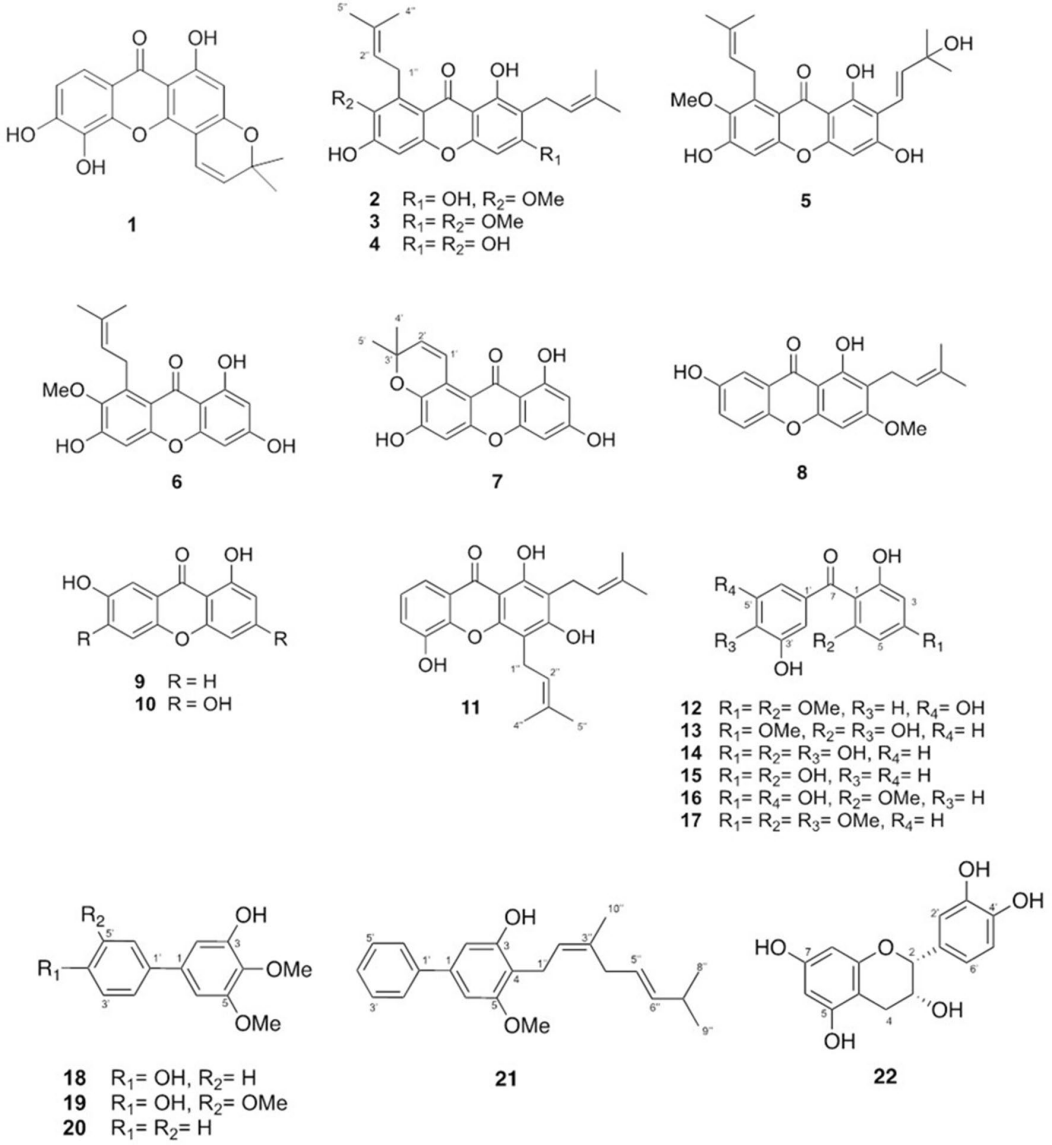
Structures of isolated compounds **1**–**22**.

Chemical investigation of EtOAc crude extract of mangosteen roots afforded two new benzophenones and one new biphenyl, namely mangostanones I–III (**12**, **13** and **18**), and one new found in naturally occurring, 4,5-dimethoxy[1,1′-biphenyl]-3-ol (**20**), along with eighteen known compounds: isojacareubin (**1**), *α*-mangostin (**2**),* β*-mangostin (**3**),* γ*-mangostin (**4**), mangostanaxanthone IV (**5**), dulxanthone D (**6**), toxyloxanthone B (**7**), 1,7-dihydroxy-3-methoxy-2-prenylxanthone (**8**), euxanthone (**9**), norathyriol (**10**), 8-deoxygartanin (**11**), maclurin (**14**), 2,3′,4,6-tetrahydroxybenzophenone (**15**), mangaphenone (**16**), (2-hydroxy-4,6-dimethoxyphenyl)(3-hydroxy-4-methoxyphenyl)methanone (**17**), garciosine A (**19**), 3-hydroxy-4-geranyl-5-methoxybiphenyl (**21**) and epicatechin (**22**) as shown in Fig. 1. The structures of the new compounds **12**, **13** and **18** were elucidated by analysis of spectroscopic data, while the known metabolites were identified by comparison of their NMR data with those reported in the literature^18–36^.

**Figure 2:**
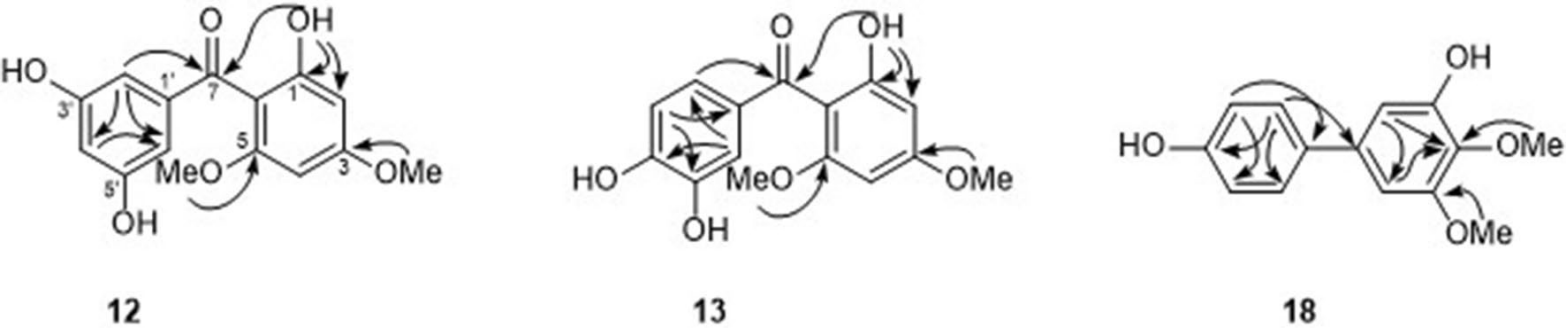
Key HMBC correlations of compounds **12**, **13** and **18**.

Mangostanone I (**1**2) was obtained as a dark-yellow solid. HRESIMS analysis showed a sodium adduct ion peak at *m/z* 313.0690 [M+Na]^+^ (calcd for 313.0688, C_15_H_14_NaO_6_). The ^1^H and ^13^C NMR spectra of compound **12** (Table 1) showed a pattern typical of a benzophenone framework^30^. The ^1^H NMR spectrum displayed signals of a 1,2,4,6-tetrasubstituted benzene ring [*δ*_H_ 6.13 (1H, d, *J* = 2.0 Hz) and 6.15 (1H, d, *J* = 2.0 Hz)], a 1,3,5-trisubstituted benzene ring [*δ*_H_ 6.60 (2H, d, *J* = 2.1 Hz) and 6.51 (1H, d, *J* = 2.0 Hz)], and two methoxy groups at *δ*_H_ 3.88 (3H, s) and 3.61 (3H, s). The ^13^C NMR spectrum assigned with the help of the HSQC data exhibited 15 signals due to two methyls, four sp^2^ methines, and six non-protonated carbons representing one carbonyl (*δ*_C_ 202.4) and five oxygenated carbons. The aromatic protons at *δ*_H_ 6.60 and 6.13 were correlated with the carbonyl group at *δ*_C_ 202.4 in the HMBC spectrum (Fig. 2), indicating the presence of a benzophenone moiety. The HMBC correlations from 4-OMe (*δ*_H_ 3.86) to C-4 (*δ*_C_ 166.2), H-3 to C-1, C-5, H-5 to C-1, C-3, and 6-OMe (*δ*_H_ 3.59) to C-6 (*δ*_C_ 162.5) confirmed the location of two methoxy units at C-3 and C-5 on ring A. From the above data, compound **1**2 was proposed structurally as shown.

**Table 1:** ^1^H and ^13^C NMR data of compounds **12, 13** and **18**.

Position	**12**^a^		**13**^a^		**18**^b^	
	δ_H_, mult., *J* (Hz)	δ_C_	δ_H_, mult., *J*(Hz)	δ_C_	δ_H_, mult., *J* (Hz)	δ_C_
1		107.9		106.2		137.4
2		163.8		163.1	6.78 d, 2.0	106.7
3	6.13 d, 2.0	94.7	6.08 s	95.8		149.5
4		166.3		161.4		134.9
5	6.15 d, 2.0	91.9	6.08 s	91.5		152.6
6		162.5		162.2	6.63 d, 2.0	103.2
7		198.4		196.1		
1′		144.0		132.8		133.9
2′	6.60 d, 2.1	106.7	7.23 d, 2.0	115.9		128.4
3′		159.1		144.3		115.7
4′	6.51 t, 2.1	105.0		149.3		155.4
5′		159.1	6.85 d, 8.4	114.2	6.87 dt, 8.0, 2.0	115.7
6′	6.60 d, 2.1	106.7	7.09 dd, 2.0, 8.4	122.7	7.42 dt, 8.0, 2.0	128.4
4-OMe	3.86 s	56.0	3.58 s	54.8	3.93 s	61.2
5-OMe					3.92 s	56.1
6-OMe	3.59 s	56.1				
3-OH					5.84 s	

^a^Measured in acetone-d_6_; ^b^measured in CDCl_3_.

Mangostanone II (**13**) was obtained as a brown-yellow solid with the molecular formula of C_15_H_14_O_6_ determined by HRESIMS at *m/z* 299.0537 [M+Na]^+^ (calcd 299.0532). Indeed, NMR spectroscopic data of **13** were similar to those of mangostanone I (**12**) (Table 1), the major difference was that compound **13** showed a resonance for a 1,3,4-trisubstituted benzene ring [*δ*_H_ 7.23 (1H, d, *J* = 2.0 Hz), 7.09 (1H, dd, *J* = 8.4, 2.0 Hz), and 6.85 (1H, d, *J* = 8.4 Hz)], while **12** had a 1,3,5-trisubstituted benzene on ring B. In addition, compound **1**2 had two methoxy groups on ring A, while compound **13** showed one methoxy at C-4. The location of the methoxy group at C-4 was indicated by the HMBC correlations of 4-OMe (*δ*_H_ 3.58) to C-4 (*δ*_C_ 161.4).

Mangostanone III (**18**) was obtained as a white powder. The molecular formula was C_14_H_14_O_4_ as determined by HRESIMS at *m/z* 269.0799 [M+Na]^+^ (calcd 269.0790). The IR spectrum of **18** showed absorption bands at 3465 cm^−1^ for free hydroxyl group, 3001 cm^−1^ for aromatic C–H stretch, and 1596 and 1576 cm^-1^ for C=C stretch. The UV spectrum showed absorption bands at *λ*_max_ at 221 and 267 nm which are typical of a hydroxygenated benzene derivative^33^. The ^1^H and ^13^C NMR spectroscopic data (Table 1) displayed two *meta*-coupled aromatic protons at *δ*_H_ 6.78 (d, *J* = 2.0 Hz, H-2) and 6.63 (d, *J* = 2.0 Hz, H-6). The remaining resonances at *δ*_H_ 7.42 (2H, dt, *J* = 8.0, 2.0 Hz, H-2′ and H-6′) and 6.87 (2H dt, *J* = 8.0, 2.0 Hz, H-3′ and H-5′) suggested a 1,4-disubstituted benzene unit on ring B. The correlation of OH-3 (*δ*_H_ 5.84, s) to C-2 (*δ*_C_ 106.7), C-3 (*δ*_C_ 149.5), and C-4 (*δ*_C_ 134.9) in the HMBC spectrum (Fig. 2) confirmed the location of hydroxyl group at C-3. The locations of the methoxy groups at C-4 and C-5 were indicated by the HMBC correlations of OCH_3_-4 to C-4 (*δ*_C_ 134.9) and OCH_3_-5 to C-5.

### Cytotoxicity of isolated compounds

To investigate anticancer properties of these compounds, preliminary screening of all isolated compounds was observed by their cytotoxic ability towards two hepatocellular carcinomas (HepG2 and Huh7) using MTT method. Sorafenib and doxorubicin were used as positive drug controls. The results (Table 2) showed that compounds **1–6** and **11** were considerable active on both cell lines tested with the IC_50_ value less than 40 μM, the compounds, except for β-(**3**) and γ-mangostin (**4**), were thus subjected to further anti-migration assay. This is because α-mangostin (**2**) with highly isolated amounts was thought to be a representative molecule of the compound series, as well as the effect of compound **3** on HCC cells has been reported^37^.

**Table 2:** Cytotoxicity of isolated compounds against HepG2 and Huh7 cells.

Compound	IC_50_ (μM) ± S.D	
	HepG2	Huh7
Sorafenib	2.66 ± 1.86	3.22 ± 1.20
Doxorubicin	3.07 ± 0.62	2.47 ± 0.59
**1**	38.30 ± 1.19	9.52 ± 1.05
**2**	5.85 ± 1.13	6.84 ± 1.28
**3**	12.43 ± 0.99	14.96 ± 1.05
**4**	11.30 ± 0.83	11.98 ± 0.68
**5**	10.43 ± 1.34	7.23 ± 1.25
**6**	22.13 ± 0.98	14.20 ± 0.90
**7**	50.04 ± 0.75	40.08 ± 0.81
**8**	> 100	> 100
**9**	> 100	50.43 ± 1.30
**10**	99.14 ± 0.66	60.8 ± 1.03
**11**	15.99 ± 1.39	9.97 ± 1.33
**12**	> 100	> 100
**13**	19.41 ± 1.19	> 100
**14**	> 100	> 100
**15**	> 100	> 100
**16**	9.81 ± 1.26	> 100
**17**	> 100	> 100
**18**	72.62 ± 0.52	87.15 ± 0.56
**19**	> 100	19.42 ± 0.88
**20**	> 100	> 100
**21**	11.87 ± 1.24	41.63 ± 1.22
**22**	> 100	> 100

### Anti-migration effects on Huh7 cells

**Figure 3:**
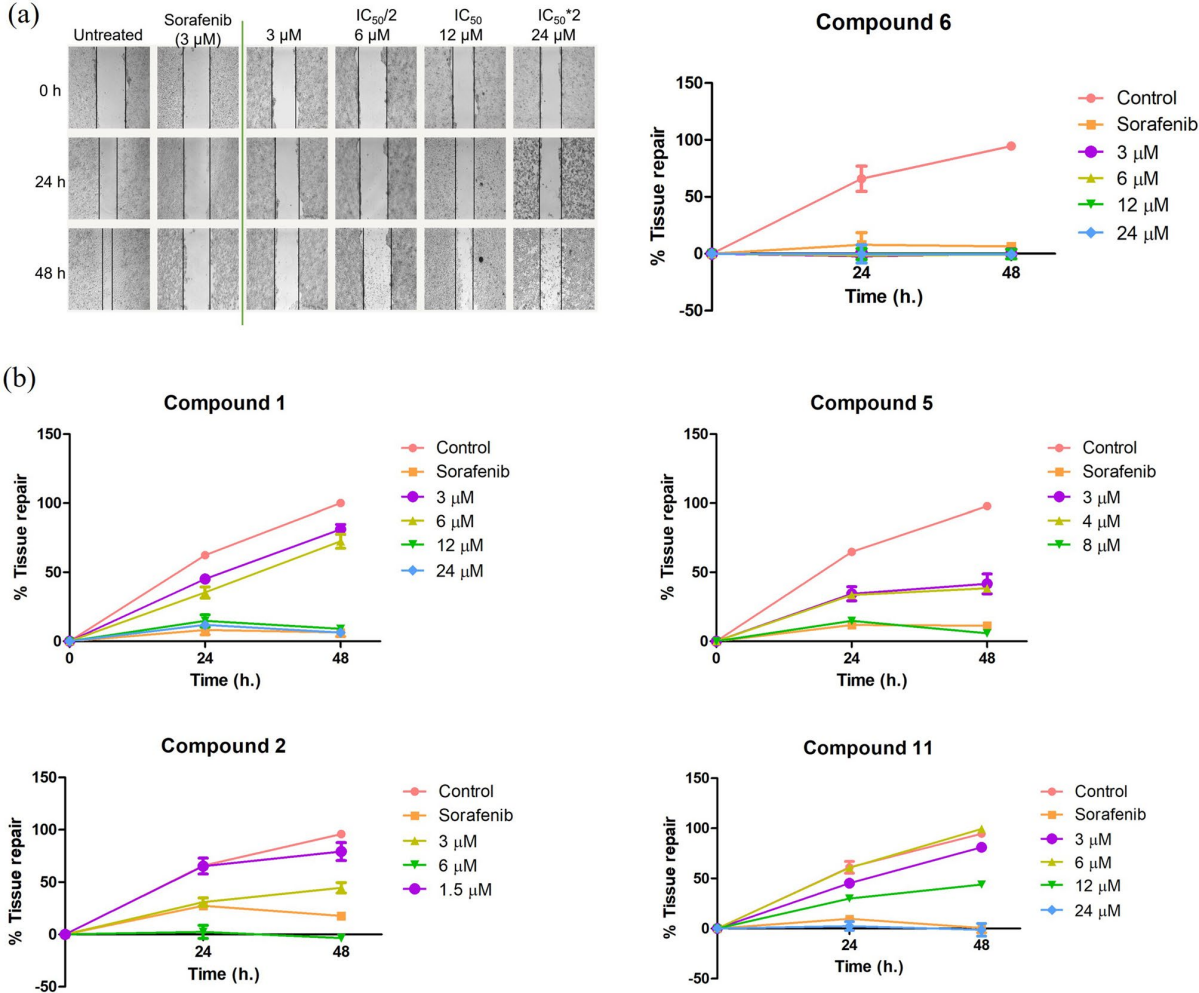
Anti-migration effect of compound **6** (**a**) and compounds **1**, **2**, **5** and **11** (**b**).

To assess whether candidate compounds could inhibit cell migration, compounds **1**, **2, 5, 6** and **11** were tested in dose- and time-dependent manner for wound healing activity using monolayer Huh7 cells scratch assay. In this experiment, the IC_50_ value of candidate compounds were considered in assessing wound healing activity. Dosage for experiments were fixed as μM at IC_50_, (IC_50_/2), (IC_50_*2), and 3 μM following the IC_50_ value of sorafenib described in previous study. Sorafenib is a standard drug approved to treat many cancer types such as hepatocellular carcinoma, renal cell carcinoma, and thyroid cancer^38^. Because of this, 3 μM were used to compare between tested compounds and standard drug in the same concentration. As a result, compound **6** showed fascinating results in inhibited cells migration. There were no increased of tissue repair percentage in all conditions, including at the same dose of sorafenib (3 μM) since 24 h (Fig. 3a). Furthermore, in higher concentration we could slightly see a change in morphology suggesting that this compound might not too toxic. Treatment of compounds **1** and **11** led to the inhibition in dose-dependent manner and the complete inhibition was observed at the high dose 24 μM without significant toxicity, whereas compounds **2** and **5** displayed moderate/weak inhibition at low concentration, but toxic at higher condition (Fig. 3b).

### Effect of compound 6 on Huh7 apoptosis

**Figure 4:**
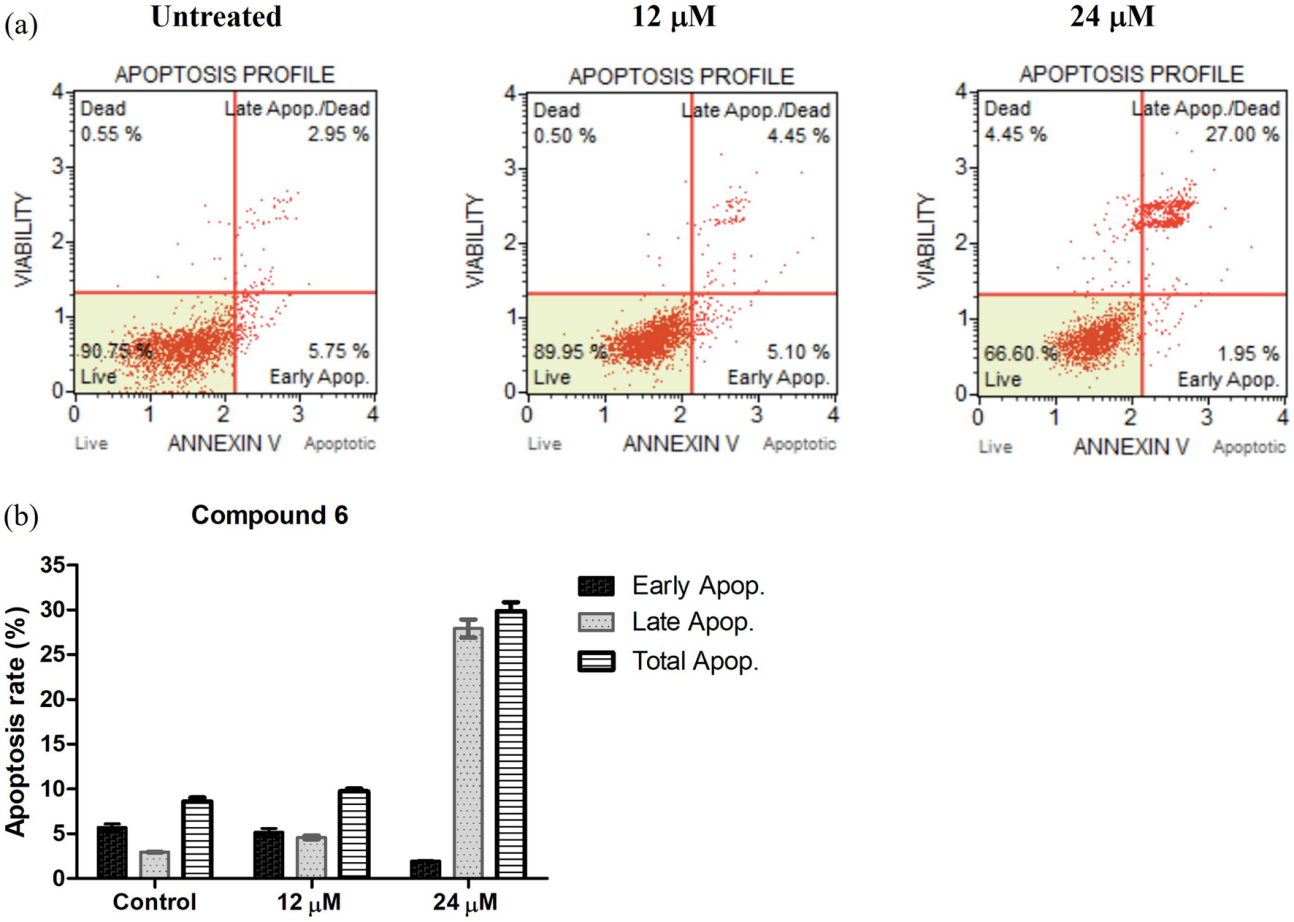
Effect of dulxanthone D (**6**) on Huh7 apoptosis**.**

Based on the above results, only compound **6** was further subjected to apoptotic assessment. The concentrations of the compound were similar to those of previous experiment (at IC_50_ and two-folds greater than IC_50_ (IC_50_*2)). Untreated cells were used as control. The Annexin V/phosphatidylserine (PS) double stain was performed to study apoptotic cells and determined by Muse^®^ Cell Analyzer. The results indicated that compound **6** was able to induce Huh7 apoptosis in dose-dependent manner, and it could be seen clearly when treated the cells with a 24 μM concentration (Fig. 4). However, the different between control and 12 μM concentration were barely seen. This result correlated with the previous experiment, explain the reason that higher concentration was way too toxic and lead to the increase of cell apoptotic.

### Effect of compound 6 on Erk and Bcl-2 family expression

**Figure 5:**
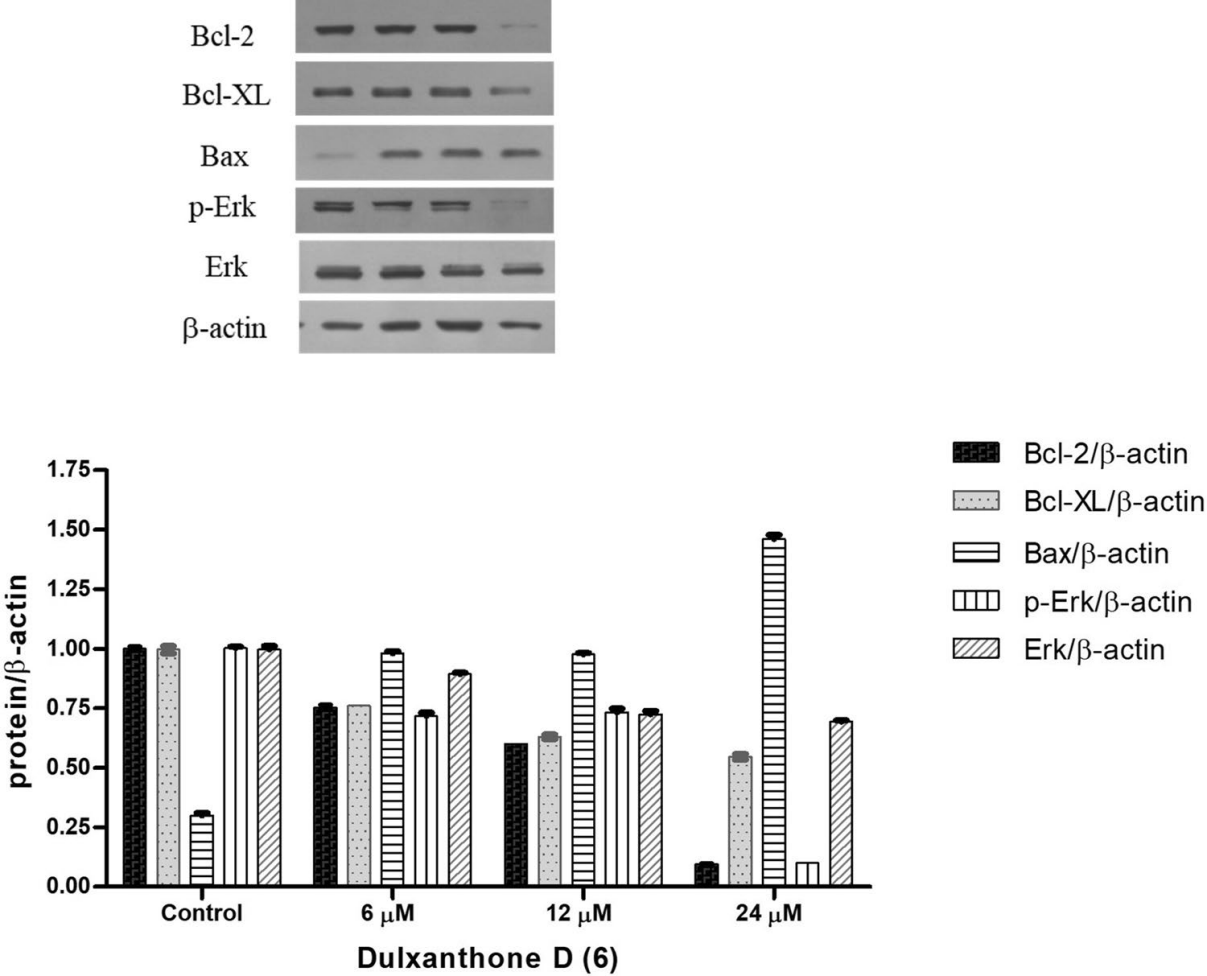
Effect of dulxanthone D (**6**) on Erk and Bcl-2 family expression.

To further observe the effect of xanthone **6** on related protein expression, Huh7 cells were treated with the compound at the same doses as previous experiments, 6, 12 and 24 μM, and the extracted protein was subjected to western blot analysis. As shown in Fig. 5, compound **6** clearly suppressed phosphorylated ERK expression in dose-dependent manner. In addition, the compound reduced the expression level of Bcl-2 and Bcl-XL and increased the level of Bax dose-dependently.

## Discussion

Xanthones are known as one of the largest constituents found in mostly parts of mangosteen. Thus, these compounds have been studies extensively in both structure elucidation and primary bioactivities. Many of them possessed medicinal abilities such as antibacterial, anti-inflammatory, and anticancer^14^. In this study, the isolated compounds obtained from mangosteen roots collected from Nakhon Sri Thammarat province, the southern Thailand are classified not only xanthones but also benzophenones and biphenyls. Among isolated compounds, three were identified as new compounds, two benzophenones and one biphenyl, namely mangostanones I–III (**12**, **13** and **18**), as well as one new naturally occurring, 4,5-dimethoxy[1,1′-biphenyl]-3-ol (**20**). The structures of the new compounds were characterized by analysis of the spectroscopic data, particularly 1D and 2D NMR and MS data. Inspired by previous studies, the cytotoxic abilities of all isolated compounds towards two hepatocellular carcinomas (HepG2 and Huh7) were evaluated using MTT assay. Sorafenib and doxorubicin were used as positive drug controls. The present study revealed that compounds **1˗6** and **11** were significantly active against both investigated cell lines with the IC_50_ values less than 40 μM. As expected, only xanthone displayed cytotoxic effect on the cancer cells tested, whereas most benzophenones and biphenyls (**12**–**21**) were inactive and/or less active. Additionally, results (Table 2) showed simple xanthones **9** and **10** did not display any significant effect. This indicated that the prenyl moiety might be required for their activity.

Cell metastasis is one of cancer cells process occurring when primary tumor spread through lymphatic and circulatory system to the nearest organs, as well as is reported to be responsible for over 90% of cancer deaths. Cell migration (motility) is a critical cause of tissue invasion supporting primary tumors to disseminate and metastasize^8–10^. Collective cell migration is mediated by coordinate cytoskeletal activity with cell–cell interaction with neighbor cells and surrounds. Therefore, it is possible to halt or retard this process at different stages by cell motility inhibitors. Of the methods available to evaluate cell migration inhibitory efficacy of the compounds, the wound-healing assay using metastatic cancer cell lines has gained popularity due to its simplicity. The assay investigates the movement of a group of cells maintaining their extracellular interaction after cells are injured by scratching and treated with selected compounds in dose- and time-depended. In this study, we performed only Huh7 cell line, which occurred *TP53* (tumor suppressor gene) mutation and represented the advance stage of HCC. We may have missed the data about the effect of compounds on anticancer activity in HepG2 cell line, which occurred TP53 wildtype and might represent the intermediate state of cancer^39,40^. To assess the cell migration inhibitory activity of the selected xanthones **1**, **2**, **5**, **6** and **11** on HCC cells, monolayer Huh7 cells were treated with the compounds at various concentrations under and above the concentration providing 50% proliferative inhibition, compared to sorafenib (3 μM). As demonstrated in Fig. 3, the xanthones apparently suppressed cell migration in dose-dependent manner, while untreated cells propagated and provided almost 100% tissue repair. Cell migration inhibition could be strikingly seen when exposed to dulxanthone D (**6**) after 24 h treatment in all conditions, including at 3 μM, the same dose of sorafenib, by the gap of injured cells being no different from the 0 h treatment. Furthermore, it was found that treatment of the compound at higher concentration or longer than 24 h did not cause seriously cell damage.

The extracellular signal-regulated kinase (ERK) signaling pathway is dominantly associated in diverse cellular processes including proliferation, survival, differentiation and motility. In addition to often up-regulation in human cancers, its multiple roles in a complex malignant lead to expect the specific blockade of the pathway results in both anti-proliferative and anti-metastatic effects in cancer cells^41^. The ERK pathway represents an attractive target for anticancer drug development. In the current study, since dulxanthone D **6** could suppress proliferation and migration of HCC cells, the effect on phophor-ERK1/2 expression was determined, compared to that of the level of ERK1/2, and **6** exerted the reduction in phophor-ERK1/2 expression as expected. This explained dulxanthone D **6** might target the components of the ERK pathway.

Apoptosis is a programmed cell death, which occur naturally in multicellular organism; however, often impaired in cancer^42^. Normally, there are two main pathways of apoptosis including intrinsic and extrinsic pathway. In this study, assessment of apoptosis was performed to analyze whether dulxanthone D **6** was able to induce apoptosis in Huh7 cell line. The results revealed **6** was able to induce Huh7 apoptosis in dose-dependent manner, clearly seen at a 24 μM concentration by 29% apoptotic rate (Fig. 4). To explore the mechanism of apoptosis induced by **6**, the investigation of the expression of apoptosis-related protein was further performed. Bcl-2 family proteins have a crucial role in promoting and inhibiting apoptosis triggered by mitochondrial dysfunction^43^. Antiapoptotic proteins, Bcl-2 and Bcl-XL, inhibit cell death by binding sequestering activator proteins, while proapoptotic proteins, including Bax, promote apoptosis. The level of Bax, Bcl-2 and Bcl-XL expression in Huh7 cells upon the treatment of dulxanthone D **6** was thus examined by western blot analysis. As a result, **6** could increase the expression level of Bax and down-regulate that of Bcl-2 and Bcl-XL dose-dependently. This indicated that dulxanthone D **6** induced apoptotic HCC cell death via the mitochondrial pathway.

## Conclusion

Phytochemical investigation of root extract of mangosteen led to the isolation and identification of twenty-two metabolites including three new compounds. Among of them, prenylated xanthones displayed the most potent cytotoxicity against HCC cells, HepG2 and Huh7. Dulxanthone D (**6**), displaying the most promising antimigration, dose-dependently induced apoptosis in Huh7 cells. Its apoptotic activation was proved to be intrinsic pathway, by involving in the decrease in Bcl-2 and Bcl-XL and the increase in Bax expression level. These findings make dulxanthone D **6** an interesting lead in the development of new anti-HCC cancer agent. Therefore, the anti-metastatic potency of **6** and its underlying mechanism in details is under further study.

## Methods

### Plant material

The mangosteen (*Garcinia mangostana*) roots were collected in May 2019 from Nakhon Sri Thammarat province, Thailand, with permission of Mrs. Narumon Choodej, the farm owner. The plant used in this study is not wild. Identification was performed by a staff of Royal Forest Department, Nakhon Si Thammarat Province. A voucher specimen was assigned with the code CUCHEM2019-003 and is deposited at Department of Chemistry, Faculty of Science, Chulalongkorn University. All methods were carried out in accordance with relevant guidelines in the method section.

### Extraction and isolation

The powdered mangosteen roots (2.4 kg) were extracted 3 times with MeOH for 3 days (5 L each per time) at room temperature. After concentrated under reduced pressure, the latter was partitioned between H_2_O and EtOAc in equal amount for 3 times. The EtOAc layer was concentrated under reduced pressure to get the EtOAc crude extract (53.08 g). The extract was chromatographed on SiO_2_ column using EtOAc:n-hexane (2:8) to give ten fractions (A-J). Fraction H (2.9 g) was separated by Sephadex LH-20 (MeOH) to gain three subfractions (H1-H3). Subfraction H1 was meticulously separated with SiO_2_ column using a mixture of acetone:n-hexane (3:7) to afford compounds **1** and **10** (3.8 and 31.7 mg respectively). Subfraction H3 was separated SiO_2_ column with a 4:6 ratio of acetone:n-hexane to yield compound **12** (5.7 mg), together with eight fractions (H3.1-H3.8). Later, subfraction H3.7 was then re-chromatographed with ODS (C-18) column using a mixture of H_2_O:MeOH (2:8) and provided compounds **13** (32.9 mg) and **14** (10.9 mg). Subfraction H2 was treated similarly to subfraction H1 to obtain compound **16** (13.3 mg) with another subfraction H2.1. The subfraction H2.1 was then subjected to ODS (C-18) column with H_2_O:MeOH (2:8) as eluent, gave out compound **15** (98.7 mg). Subsequently, Fraction E (2.6 g) was separated by Sephadex LH-20 column with MeOH and give six subfractions (E1-E6) which E3 was crystalized and yielded compound **3** (281 mg). Fraction E5 was further purified by reversed-phase ODS (C-18) column using H_2_O:MeCN (2:8) mixture to obtain compound **2** (301.6 mg). Following by fraction C (346.9 mg), was subjected to SiO_2_ column utilizing acetone:n-hexane (1:9) to obtain six subfractions (C1-C6). Subfraction C4 then was subjected to SiO_2_ column using CH_2_Cl_2_:n-hexane gradient from 6:4 to 9:1 and gave compounds **5** and **20** with weight 5.8 and 120.2 mg, respectively. Fraction B was separated by SiO_2_ column using EtOAc:n-hexane (1:9) mixture to give nine subfractions (B1-B9). Subfraction B4 was picked to separate with Sephadex LH-20 using MeOH and gave out compound **21** (58.8 mg). For fraction D, the chromatography was performed using SiO_2_ column with CH_2_Cl_2_:n-hexane from 7:3 to 9:1 gradient elution to yield fifteen subfractions (D1-D15). Subfractions D4 was subjected to Sephadex LH-20 column (MeOH), followed by SiO_2_ column with EtOAc:n-hexane (2:8) mixture to yield compound **9** (5.8 mg). Subfractions D6 was separated by SiO_2_ column with acetone:n-hexane (2:8) mixture to give nine fractions (D6a-D6i). D6d was further purified by to Sephadex LH-20 eluting by MeOH, followed by SiO_2_ column with a gradient of EtOAc:n-hexane (1:9 to 2:8) to yield compound 11 (7.8 mg). Subfraction D14 was separated by ODS column with a 2:8 mixture of H_2_O:MeOH (2:8) to give compound **8** (1.3 mg). Furthermore, fraction G was subjected with acetone:n-hexane (2:8) elution on SiO_2_ column to obtain ten subfractions (G1-G10). Combined G6 and G7 fraction was then purified by ODS (C-18) column with H_2_O:MeOH (1:9) to yield compound **17** (30.4 mg). Fraction J was eluted over Sephadex LH-20 with MeOH to obtain seven subfractions (J1–J7) and one pure compound (22, 15.8 mg). Subfraction J3 was further chromatographed with SiO_2_ column utilizing acetone:n-hexane (4:6), and, lastly, ODS (C-18) column chromatography of subfraction J3e afforded compound **4** (3.6 mg). Fraction F was subjected to SiO_2_ column utilizing EtOAc:CH_2_Cl_2_ gradient from 0:10 to 1:9 to obtain ten subfractions (F1-F10). Compound **19** (60 mg) was obtained by separating subfraction F7 with SiO_2_ column using acetone:n-hexane (3:7) together with impure compound **18**. The impure compound was further purified by ODS (C-18) column utilizing H_2_O:MeCN (2:8) to yield pure compound **18** (31.4 mg). Subfraction F10 was isolated by Sephadex LH-20 using MeOH to afford compound **7** (1.9 mg) and another five subfractions (F10a–F10e). Fraction F10b was subjected to ODS (C-18) column eluted with H_2_O:MeOH (3:7) to provide compound **6** (3.9 mg).

### Structure elucidation of isolated compounds by spectroscopic techniques

^1^H, ^13^C, and 2D NMR spectroscopic data were obtained from Bruker AV400 spectrometer (400 MHz for ^1^H NMR, 100 MHz for ^13^C). Deuterated CDCl_3_ and acetone-d_6_ were used as NMR solvent. HRESIMS data was obtained with a Bruker micrOTOF. FT-IR data was obtained from Perkin-Elmer Model 1760X spectrophotometer.

### Cell culture

Human Hepatocellular Carcinoma (HCC) cell lines, HepG2 (JCRB1054) and Huh7 (JCRB0403) were purchased from Japanese Collection of Research Bioresources (JCRB), National Institutes of Biomedical Innovation, Health and Nutrition, Osaka, Japan. Both were cultured in Dulbecco’s Modified Eagle’s medium (DMEM) supplemented with 10% fetal bovine serum (FBS) and 1% penicillin/streptomycin at 37 °C in a 5% CO_2_ humidified incubator.

### Cytotoxicity assay

Cytotoxicity activity was performed using MTT assay. Briefly, HCC cells were seeded in 96-well plate with a density of 1 × 10^4^ cells/well. Then, the cells were incubated at 37 °C with 5% CO_2_ for 24 h. After removed the medium supernatant, the cells were treated for 48 h with serial dilution of the tested compounds along with sorafenib and doxorubicin as standard drug control in the presence of serum-free DMEM media. MTT solution (0.5 mg/ml DMEM) was added to each well and then incubated for 4 h in a humidified atmosphere. After the incubation period, the supernatant was removed and DMSO was added to dissolve formazan crystals. The absorbance was measured at 570 nm using microplate reader. The results were calculated by using GraphPad Prism™ (version 8.0) and presented as the percentage of cells viability and half-maximal inhibitory concentration (IC_50_).

### Anti-migration assay

Wound healing assay was performed to identify cell migration. Huh7 monolayer cells were prepared in 12-well plate with a density of 5.5 × 10^5^ cells/well. The wound was created by scratching with micropipette tip (1 mm diameter). The medium was removed and replaced with serum-free DMEM containing compound 1, 2, 3, 4, 7, 8, and 21 (at IC_50_, < IC_50_ and > IC_50_ concentrations for each compound). Sorafenib (3 μM) was used as a positive control and cells treated only with serum-free medium were used as standard control. The cells were observed and photographed using microscope at 0, 24, and 48 h. The wound healing progression was calculated and presented as percentage of tissue repair (%Tissue repair).

$$ \% {\text{Tissue repair}} = \frac{{{\text{Wound space}}_{{0{\text{ h}}}} - {\text{Wound space}}_{{\text{n h}}} }}{{{\text{Wound space}}_{{0{\text{ h}}}} }} \times 100 $$

where Wound space_0 h_: length of gap between scratched cells (μm) at 0 h.

Wound space_n h_: length of gap between scratched cells (μm) at n h.

### Apoptosis assessment

Apoptosis was determined by measuring external phosphatidylserine, which located on the outer of cell membrane. Huh7 cells were seeded in 24-well plate with a density of 2.5 × 10^5^ cells/well and incubated for 24 h with 5% CO_2_ at 37 °C. Cells were treated with compound **6** (using IC_50_ value and 2 times above of IC_50_ value) for 24 h. Then, cells were detached with trypsin and collected to wash with PBS and resuspended in 1 ml of DMEM. Then, Annexin V-7AAD reagent (Luminex Corp., Austin, TX, USA) was added in the cell’s suspension with 1:1 ratio and stained for 30 min at room temperature in the dark. Live, dead, and apoptosis cell populations were determined using Muse^®^ Cell Analyzer. The results were analyzed and calculated as apoptosis profile.

### Western blot assay

After treated Huh7 with compound **6** at IC_50_ concentration and double in value for 24 h with density of 2.5 × 10^5^ cells/well in 24 wells-plate, cells were harvested at time-depended and total protein were collected using ice cold lysis buffer. Protein samples then calculated and loaded in SDS-PAGE to separate using electrophoresis. Further, protein was transferred from gel to polyvinylidene fluoride (PVDF) transmembrane and blocked with 5% skimmed milk. The primary antibodies then added and incubated for 24 h in 4 °C. Membrane was washed with PBST afterward, then secondary antibodies were added and incubated with in optimal times. Membrane was washed again with PBST and performed color developer to visualize in the dark room. The results were photographed and analyze using Image J program.

### Statistical analysis

Data are presented as mean ± SD values. GraphPad prism™ ver. 8 program with one-way analysis of variation was used for testing the significance, *p* < 0.05 was considered as statistically significance.

## Supplementary Information

Supplementary Information.

## Data Availability

All data generated or analyzed during this study are included in this article.
