# Supplementary material for: Bioactive xanthones, benzophenones and biphenyls from mangosteen root with potential anti-migration against hepatocellular carcinoma cells
Source: Sci Rep. 2022 May 21;12:8605. doi: 10.1038/s41598-022-12507-8 (PMC9124209; doi:10.1038/s41598-022-12507-8)

Supplementary Information

Bioactive xanthones, benzophenones and biphenyls from mangosteen root with potential anti-migration against hepatocellular carcinoma cells

Siwattra Choodej[1], Kedkarn Koopklang[2], Achara Raksat[1], Natthaya Chuaypen[3], Khanitha Pudhom[1,*]

[1]Department of Chemistry, Faculty of Science, Chulalongkorn University, Bangkok 10330, Thailand. [2]Program in Biotechnology, Faculty of Science, Chulalongkorn University, Bangkok 10330, Thailand. [3]Center of Excellence in Hepatitis and Liver Cancer, Department of Biochemistry, Faculty of Medicine, Chulalongkorn University, Bangkok 10330, Thailand. Email: khanitha.p@chula.ac.th

Supplementary information available

**Fig. S1.**	NMR data (acetone-d_6) of compound **12** (a) ^{1}H NMR spectrum (400 MHz); (b) ^{13}C NMR spectrum (100 MHz); (c) ^{1}H-^{1}H COSY spectrum; (d) HSQC spectrum; (e) HMBC spectrum
**Fig. S2.**	NMR data (acetone-d_6) of compound **13** (a) ^{1}H NMR spectrum (400 MHz); (b) ^{13}C NMR spectrum (100 MHz); (c) ^{1}H-^{1}H COSY spectrum; (d) HSQC spectrum; (e) HMBC spectrum
**Fig. S3.**	NMR data (acetone-d_6) of compound **18** (a) ^{1}H NMR spectrum (400 MHz); (b) ^{13}C NMR spectrum (100 MHz); (c) ^{1}H-^{1}H COSY spectrum; (d) HSQC spectrum; (e) HMBC spectrum
**Fig. S4.**	Wound healing assay of compounds **1**, **2, 5** and **11**.
**Fig. S5.**	The whole images representing Bcl-2, BCl-XL and Bax (a), p-Erk1/2 and Erk (b) with color prestained protein standard.

Fig. S1. NMR data (acetone-d$_6$) of compound **12** (a) ^{1}H NMR spectrum (400 MHz); (b) ^{13}C NMR spectrum (100 MHz); (c) ^{1}H-^{1}H COSY spectrum; (d) HSQC spectrum; (e) HMBC spectrum

(a)

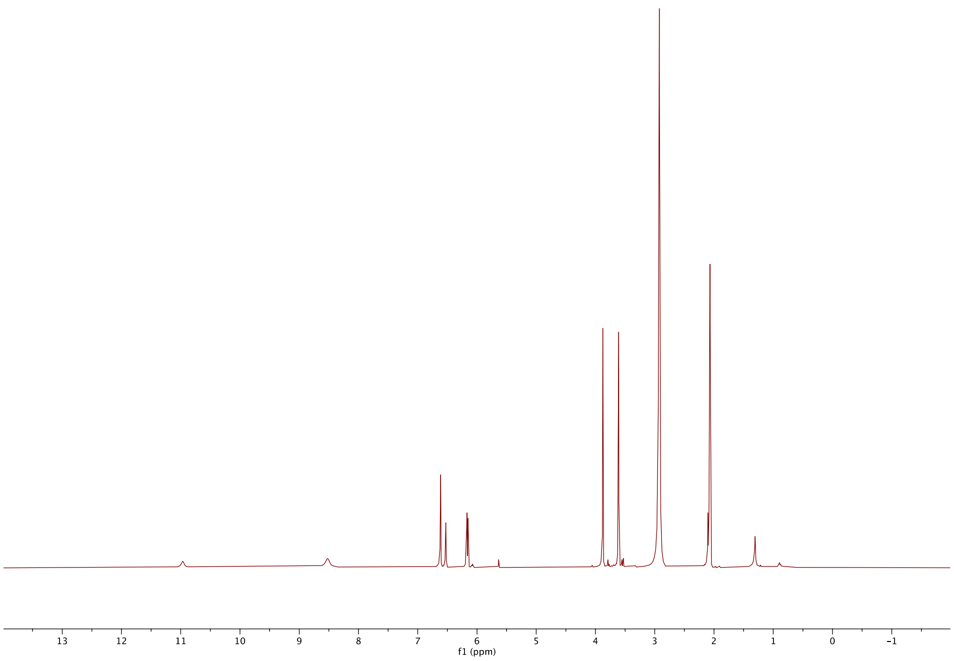

(b)

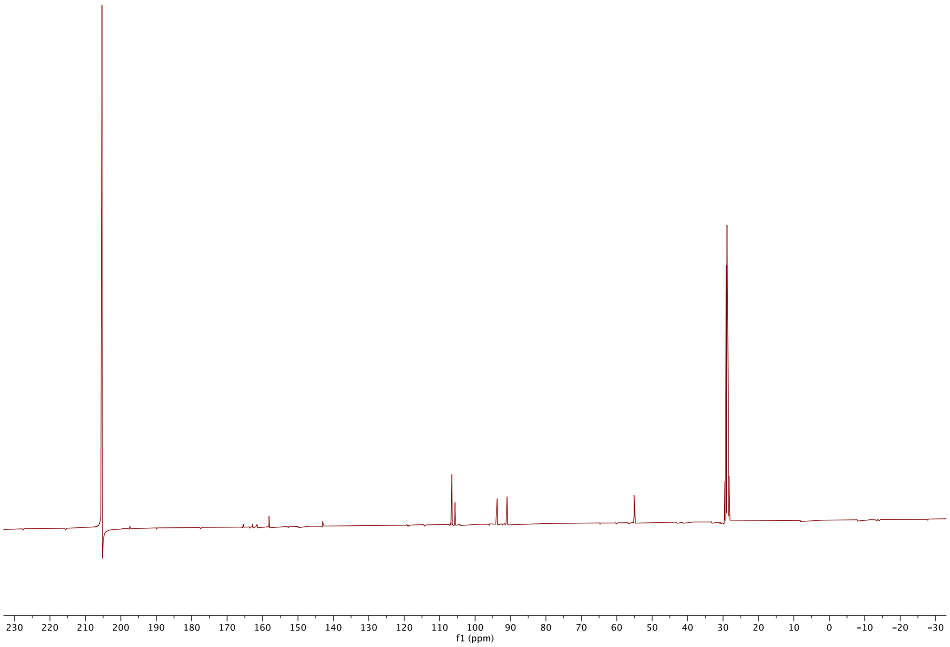

(c)

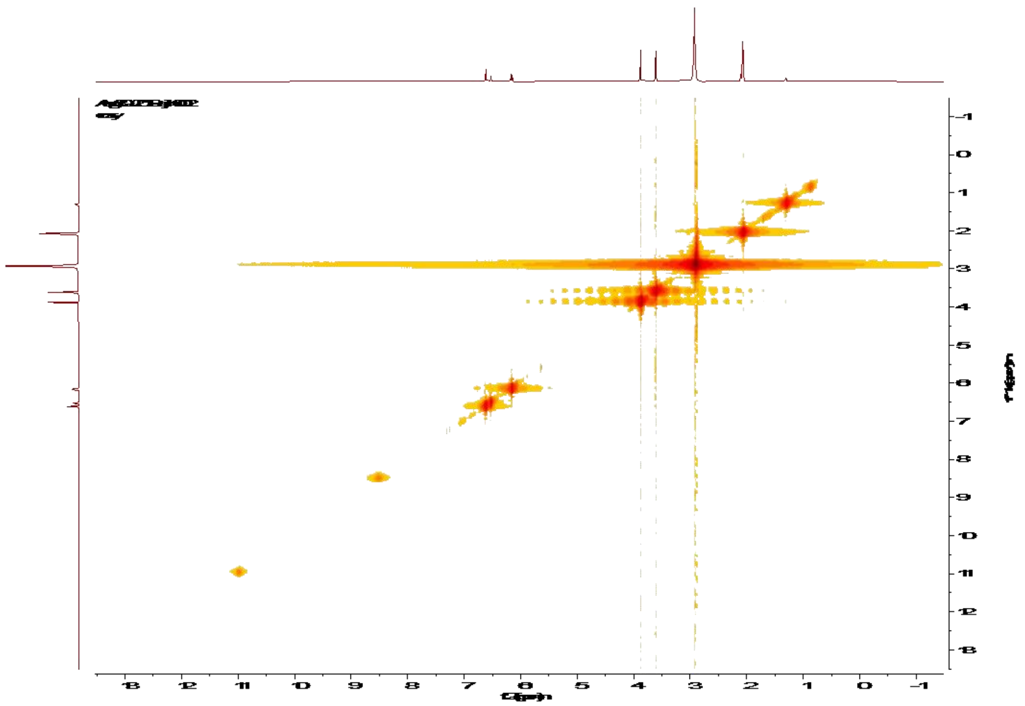

(d)

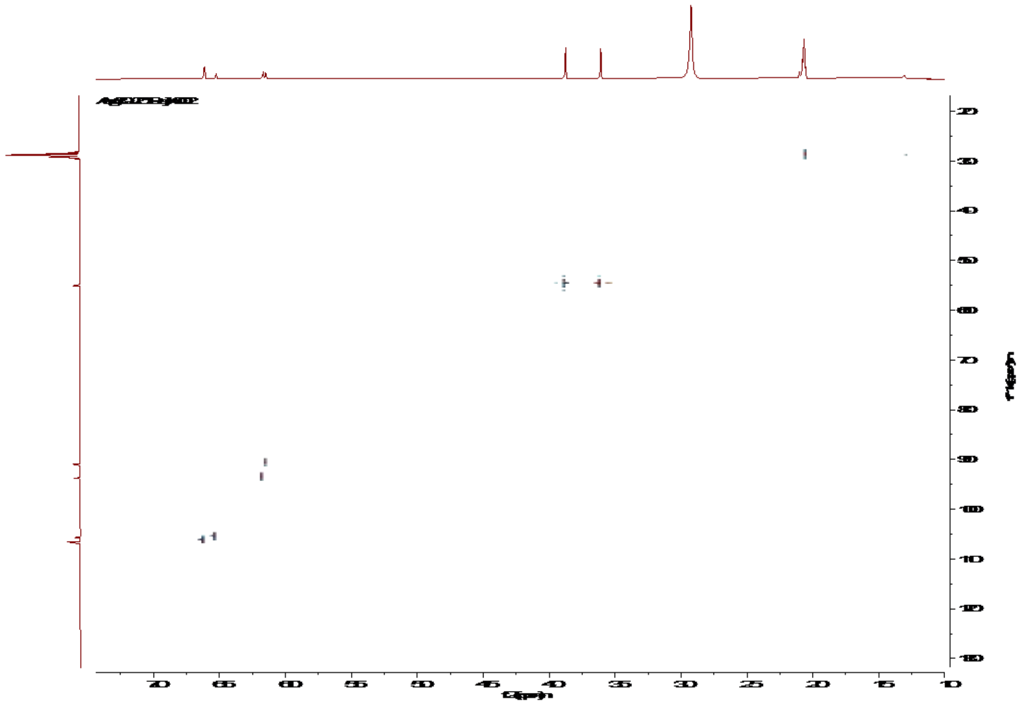

(e)

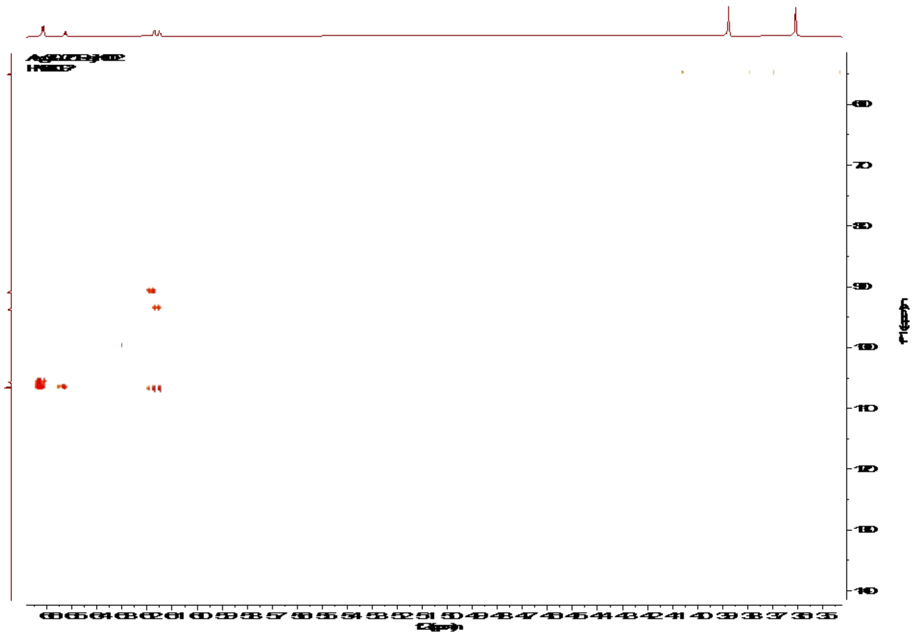

Fig. S2. NMR data (acetone-d$_6$) of compound **13** (a) ^{1}H NMR spectrum (400 MHz); (b) ^{13}C NMR spectrum (100 MHz); (c) ^{1}H-^{1}H COSY spectrum; (d) HSQC spectrum; (e) HMBC spectrum

(a)

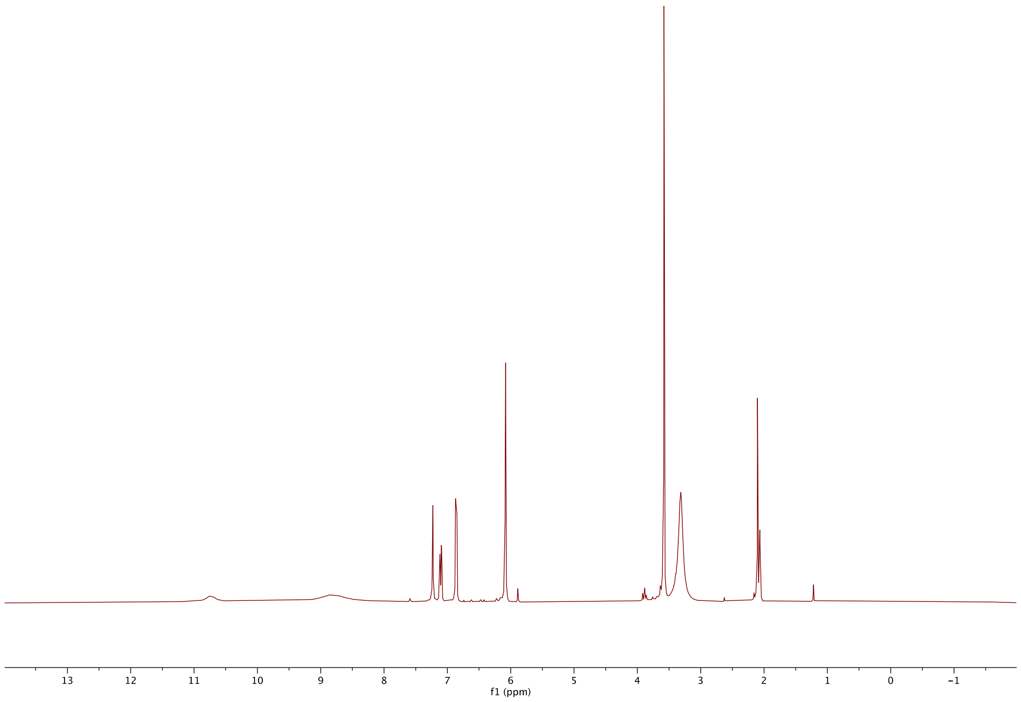

(b)

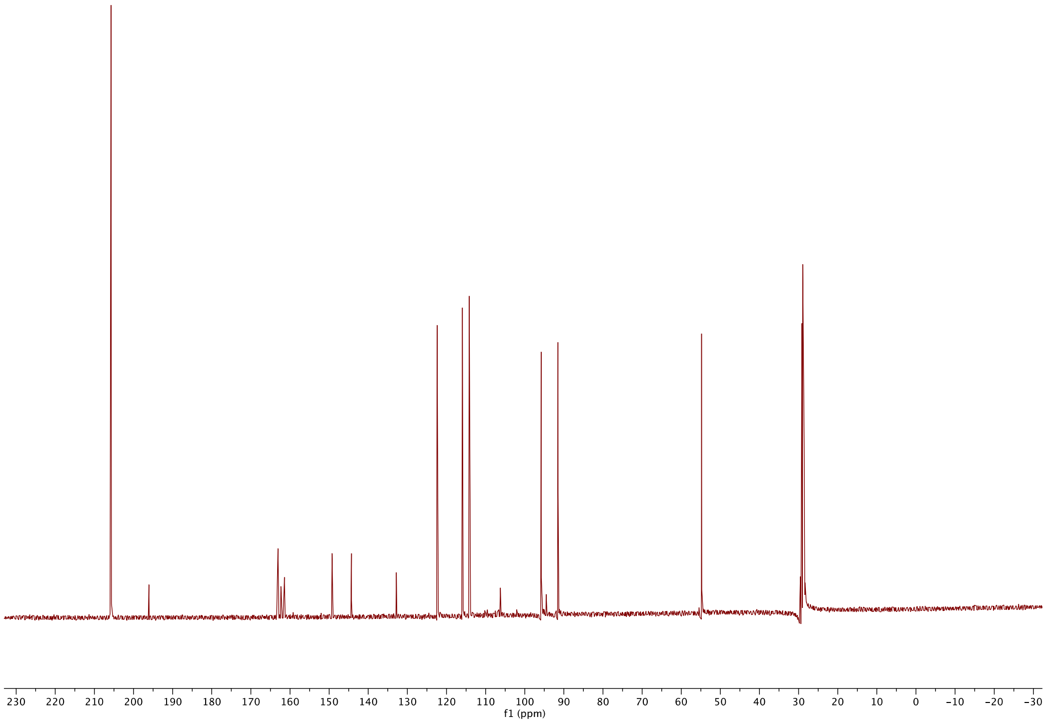
230 220 210 200 190 180 170 160 150 140 130 120 110 100 90 80 70 60 50 40 30 20 10 0 −10 −20 −30
f1 (ppm)

(c)

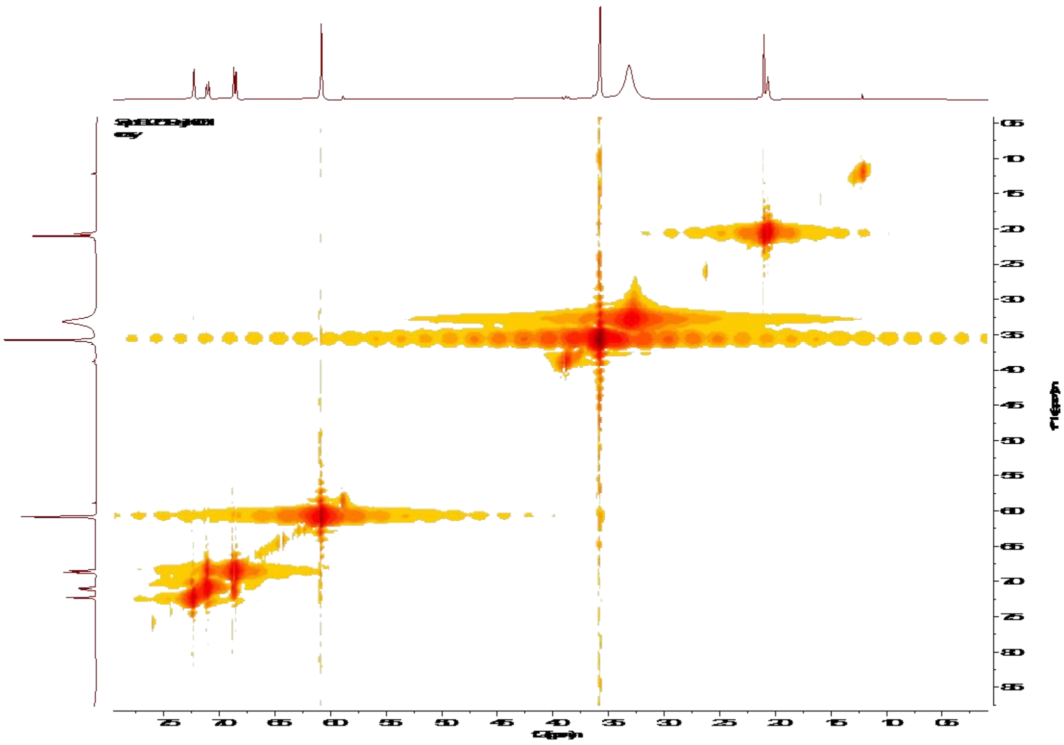

(d)

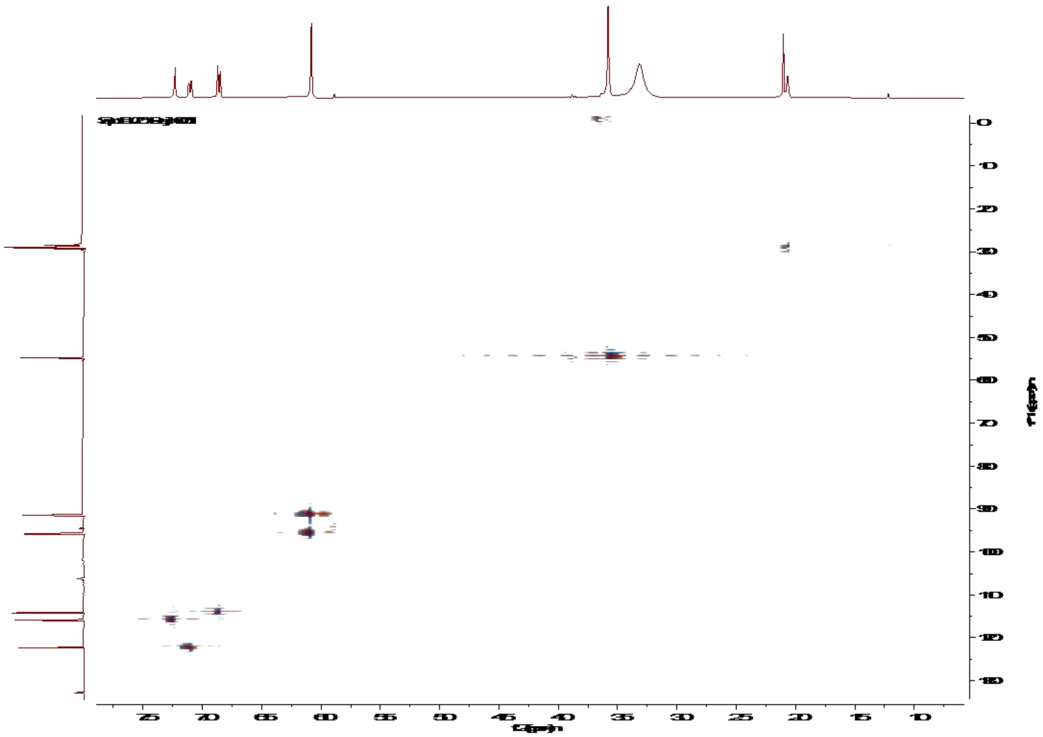

(e)

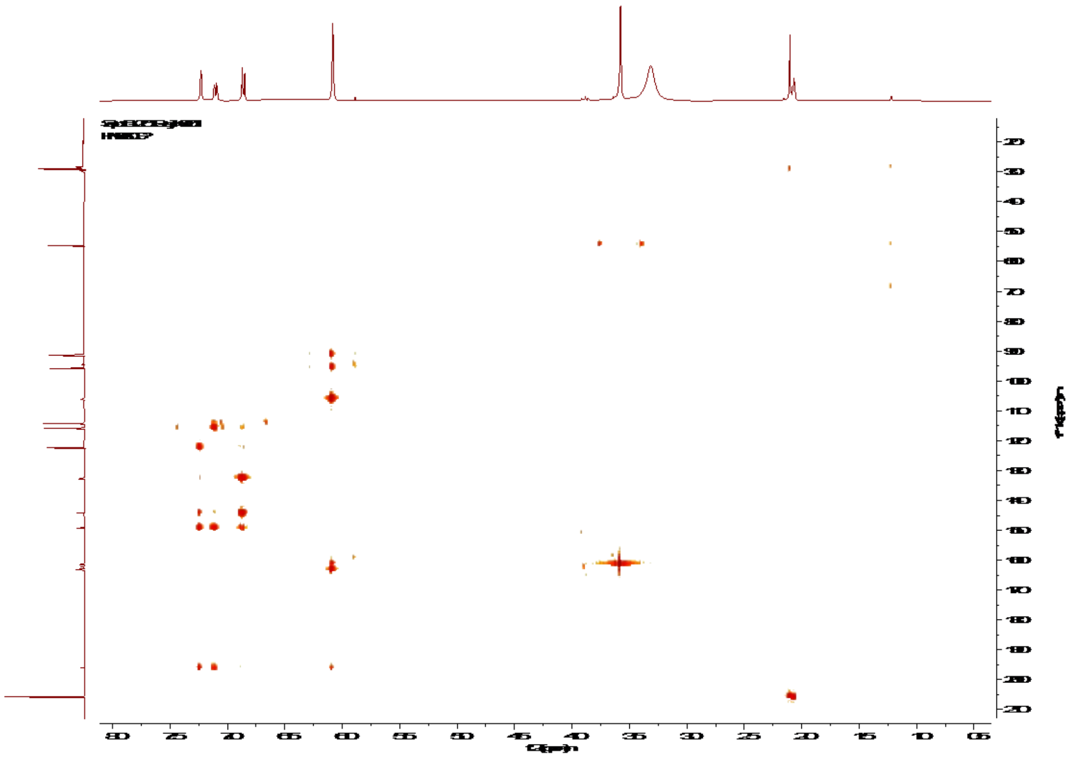

Fig. S3. NMR data (acetone-d$_6$) of compound **18** (a) ^{1}H NMR spectrum (400 MHz); (b) ^{13}C NMR spectrum (100 MHz); (c) ^{1}H-^{1}H COSY spectrum; (d) HSQC spectrum; (e) HMBC spectrum

(a)

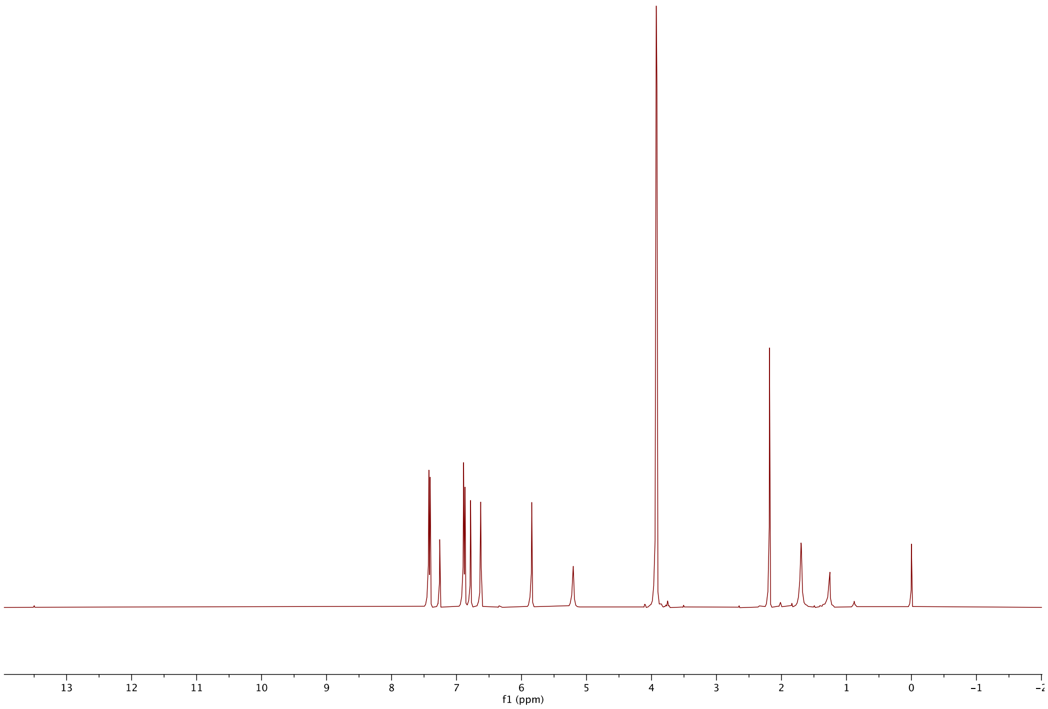

(b)

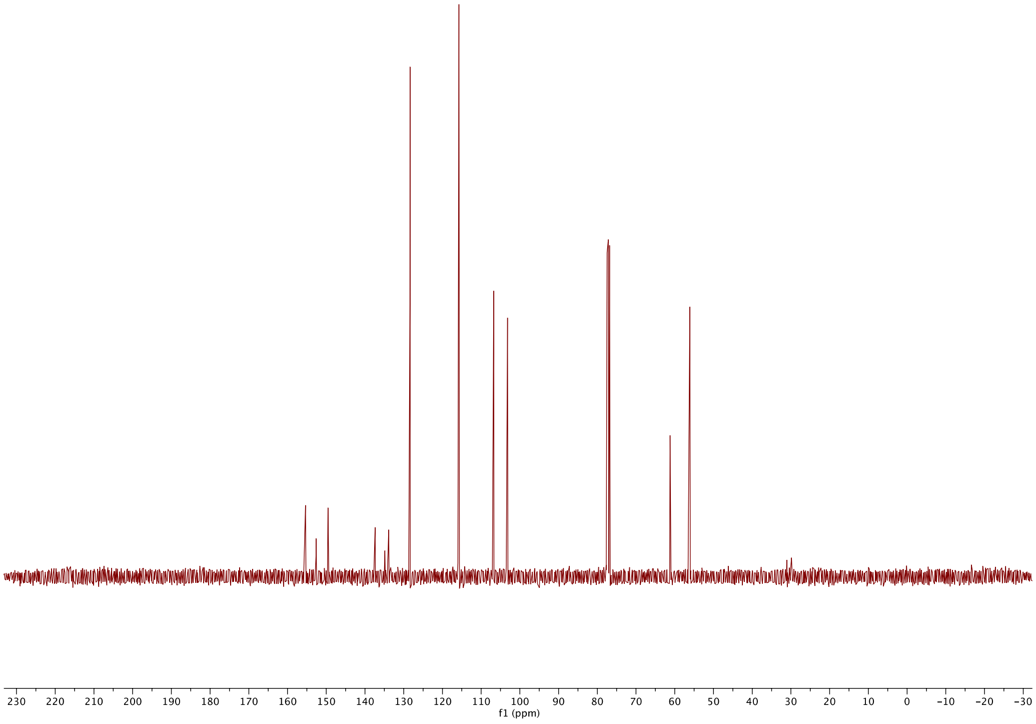

(c)

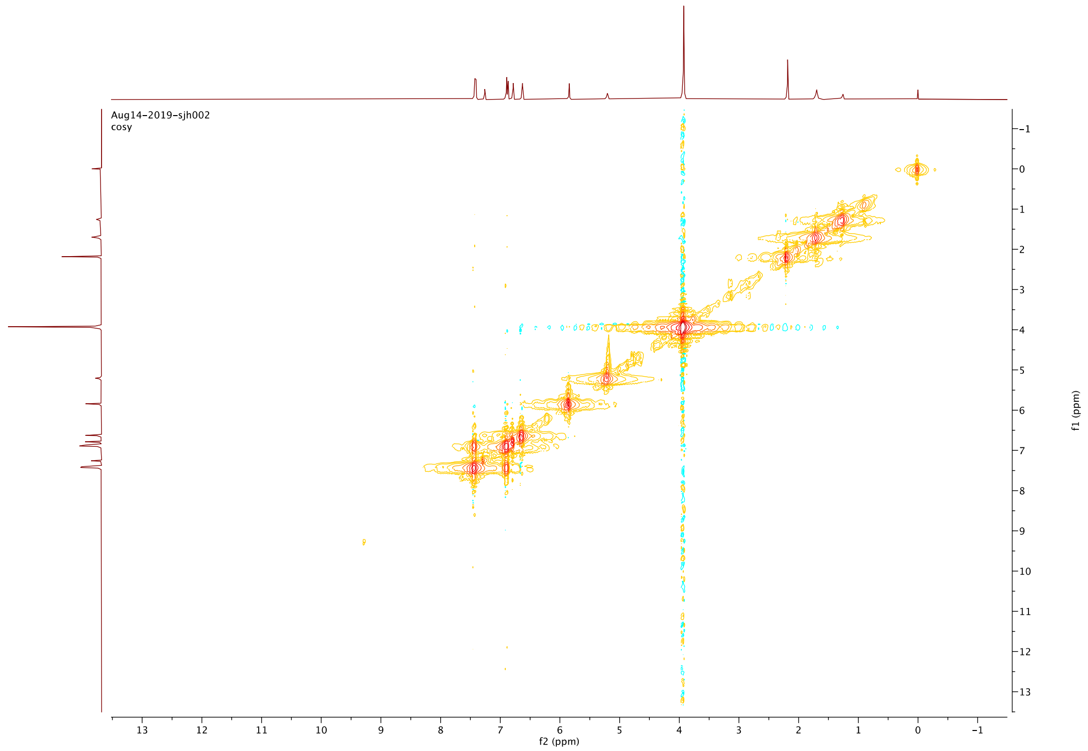
Aug14-2019-sjh002
cosy
f2 (ppm)
f1 (ppm)

(d)

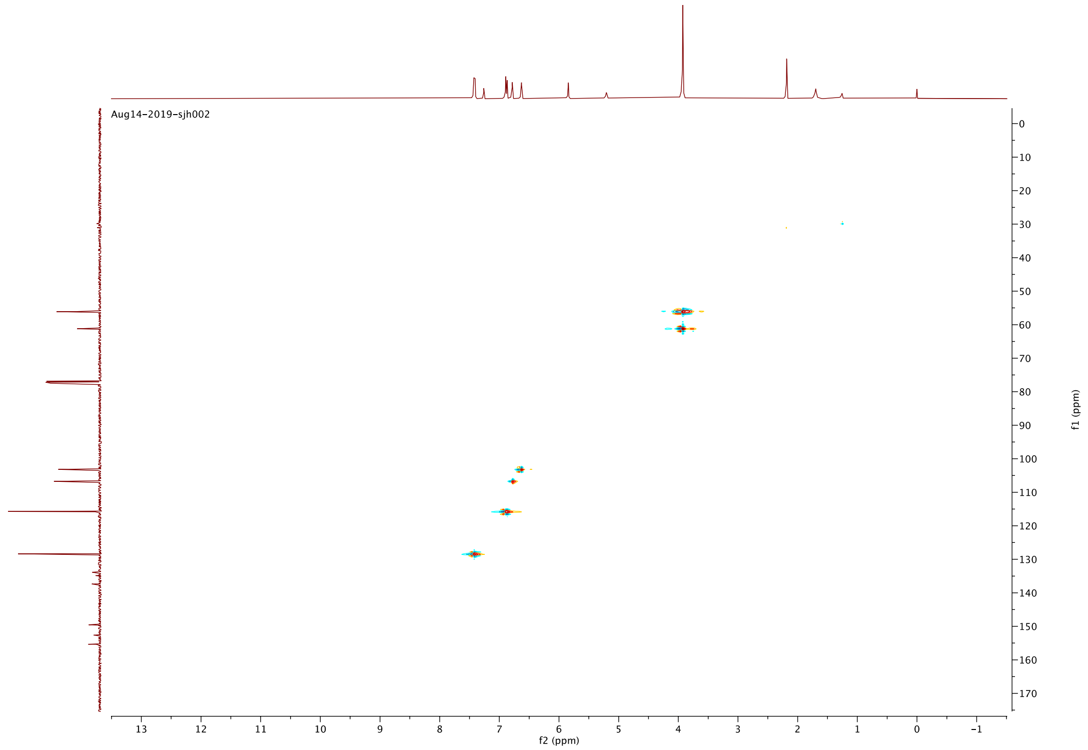
Aug14-2019-sjh002
f2 (ppm)
f1 (ppm)

(e)

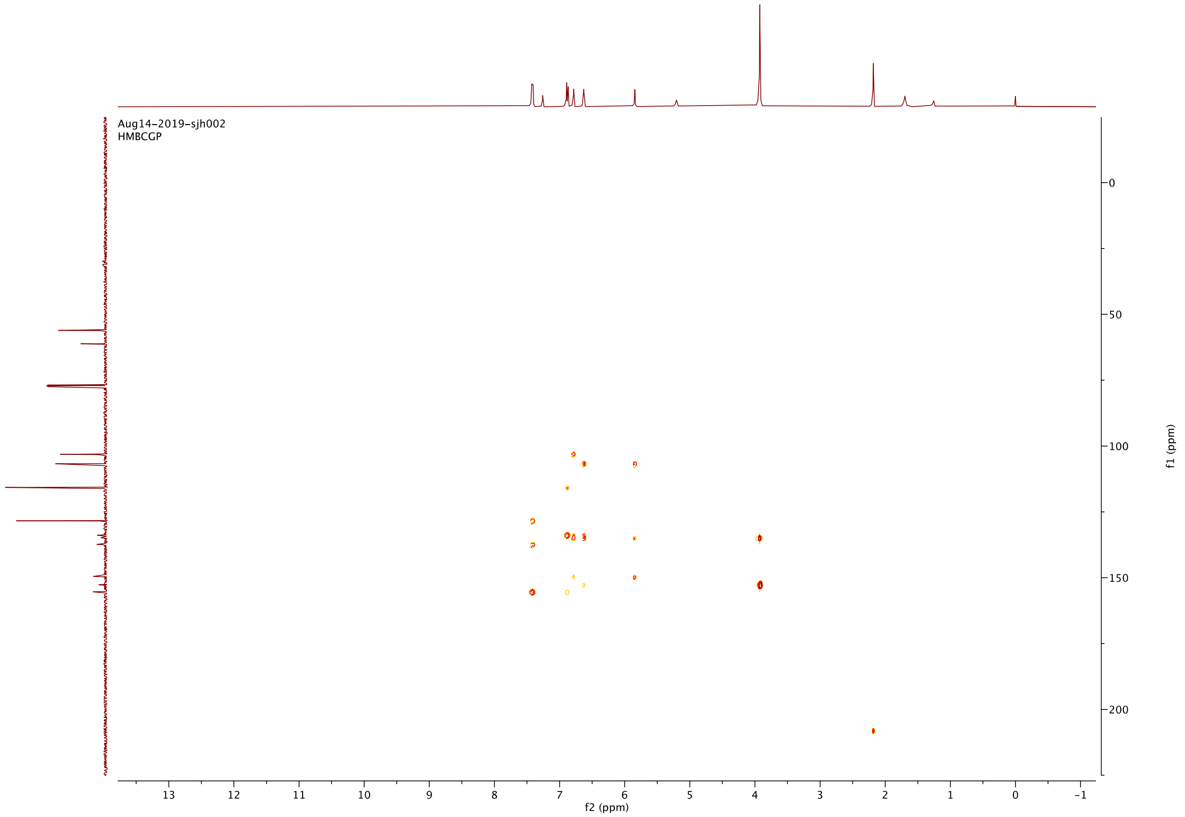

Fig. S4. Wound healing assay of compounds **1**, **2, 5** and **11**.

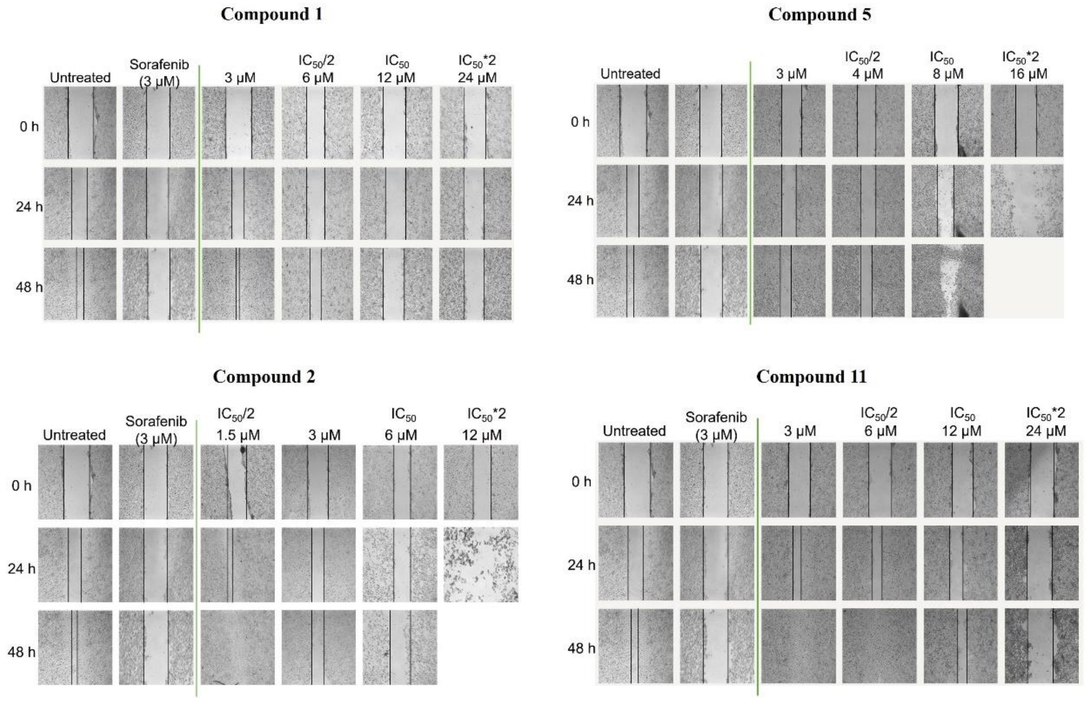

Fig. S5. The whole images representing Bcl-2, BCl-XL and Bax (a), p-Erk1/2 and Erk (b) with color prestained protein standard. Remark: All black blocks in figure are made due to the bands from other compound which is not related to this research.

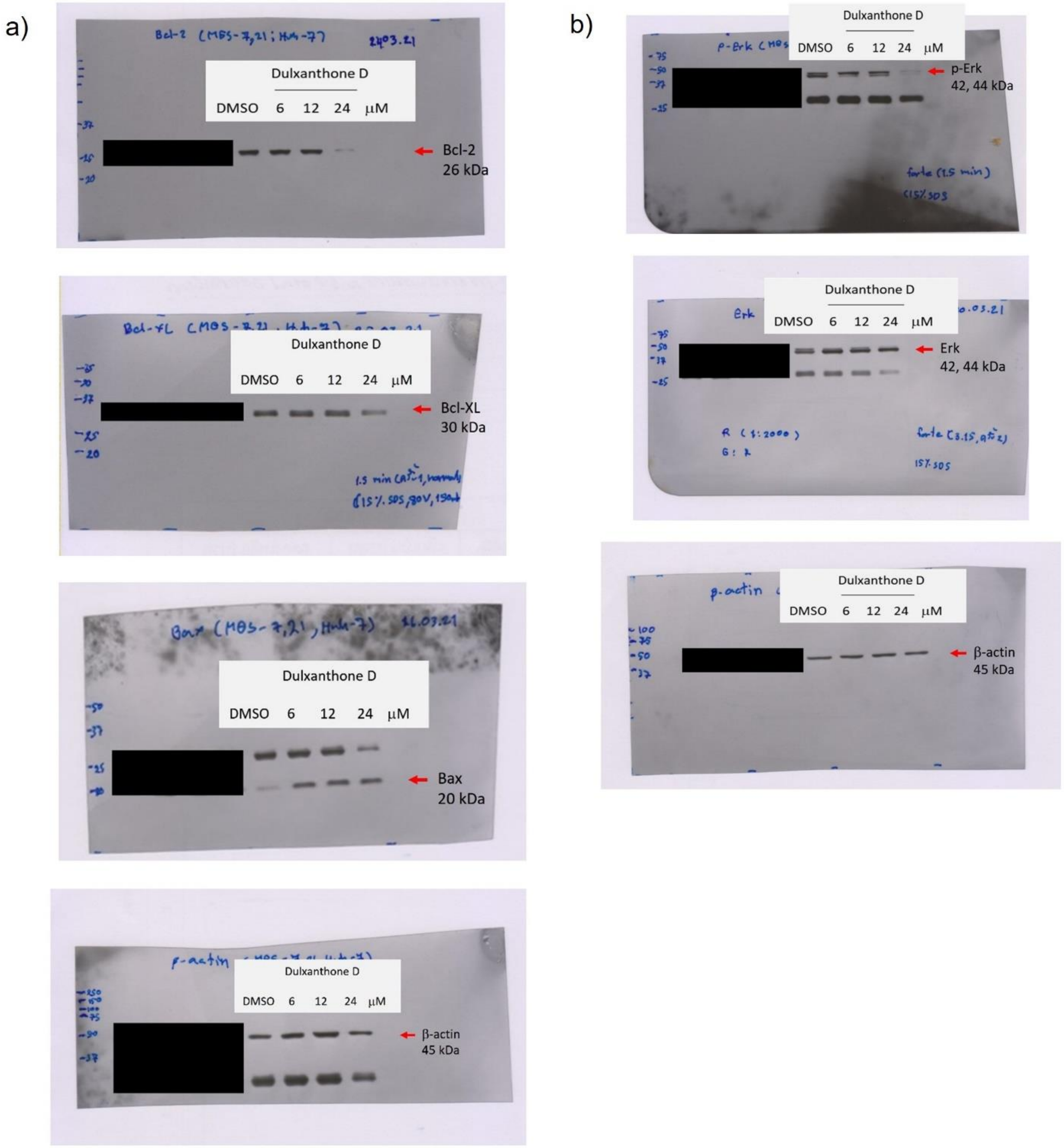

Supplement: Supplementary file 1 — Supplementary Information. [file 41598_2022_12507_MOESM1_ESM.pdf]
